# Lymphedema Management in May–Thurner Syndrome: A Case Series of 26 Patients

**DOI:** 10.3390/jcm15197546

**Published:** 2026-09-28

**Authors:** Sabrina Valentina Lazar, Elizabeth Tadevosyan, Nitya Devisetti, Subin Cho, Sirish Kishore, Carrie Kubiak, Dung Hoang Nguyen

**Affiliations:** 1 Department of Surgery, University of Massachusetts Chan Medical School Program, Worcester, MA 01655, USA; 2 Division of Plastic and Reconstructive Surgery, Stanford University School of Medicine, Palo Alto, CA 94304, USA; 3 Rutgers New Jersey Medical School, Newark, NJ 07103, USA; 4 Department of Radiology, Stanford University School of Medicine, Palo Alto, CA 94305, USA; 5 Department of Plastic Surgery, Vanderbilt University Medical Center, Nashville, TN 37232, USA

**Keywords:** May–Thurner syndrome, lymphedema, liposuction, lymphovenous bypass, vascularized lymph node transfer

## Abstract

**Background**: May–Thurner syndrome (MTS) is an uncommon cause of deep vein thrombosis and unilateral lower extremity lymphedema. We present the largest case series to date of 26 MTS patients with lymphedema. We aim to report the pathophysiology of lymphedema in relation to venous dysfunction onset in MTS patients and evaluate the effectiveness of surgical lymphedema treatment. **Methods**: Retrospective case series. Patients treated for lymphedema with concurrent MTS diagnosis were retrospectively identified at a large academic medical center. Surgical interventions included lymphovenous anastomosis (LVA), vascularized lymph node transfer (VLNT), liposuction, and a combination of surgeries. Nonsurgical intervention included complete decongestive physical therapy (CDPT). **Results**: Twenty-six patients (19 female, seven male) were identified and had undergone prior iliac vein stenting. International Society of Lymphology (ISL) stage II–III patients underwent lymphatic surgery or CDPT, while stage I only underwent CDPT. Stage I patients demonstrated an average excess volume increase of 21.74% ± 23.11. In stage II, CDPT patients experienced a percent volume increase of 15.26% ± 11.06, while combination patients experienced a 102% ± 63.31 volume decrease. Stage III patients comprised CDPT, liposuction, VLNT, and combination; these groups and two of three VLNT patients demonstrated decreased limb volume percentage. Pre- and postoperative MRIs in surgically treated patients showed improved tissue bulk, edema reduction, and new lymphatic channel formation. **Conclusions**: Surgery was effective in reducing MTS-related lymphedema. The common co-occurrence of lymphedema and MTS presents an opportunity for screening, early intervention, and prospective studies.

## 1. Introduction

May–Thurner syndrome (MTS) or iliac vein compression syndrome is an uncommon cause of deep vein thromboses (DVTs) and lower extremity lymphedema. This disorder typically results from compression of the left iliofemoral vein by the right common iliac artery against the lumbar spine, but other anatomic variants also exist [1]. MTS is associated with a range of chronic and recurrent comorbidities, such as varicosities, venous stasis changes, venous hypertension, venous ulcers, valvular dysfunction, DVT, dermatitis, hyperpigmentation, cellulitis, and lower extremity lymphedema [2,3,4]. Infrequently, MTS can even lead to life-threatening conditions, such as pulmonary embolism (PE) and phlegmasia cerulea dolens [3,4,5]. The most common symptoms of MTS are left-sided lower extremity DVT, pain, swelling, and lymphedema [4,6].

Currently, the true prevalence of symptomatic MTS is unclear. When MTS symptoms like DVT or lymphedema do occur, they are often in the context of additional risk factors, such as pregnancy, contraceptive use, malignancy, trauma, surgery, or primary lymphedema [6,7,8], which may obscure the identity of the underlying condition. Diagnostic venography with intravascular ultrasound is currently the gold standard for imaging [3,6], allowing for both visualization of vascular compression and measurement of pressure gradients across areas of iliofemoral stenosis [9]. Less-invasive imaging modalities include magnetic resonance venography (MRV), computed tomography venography (CTV), and Doppler ultrasound.

Currently, the mainstay of MTS treatment is stent placement in the iliofemoral vein at areas of obstruction [10]. Stenting may be accompanied by angioplasty; however, angioplasty alone is generally considered inadequate as a treatment for MTS [3,11]. Outcomes of stent placement appear favorable, with high rates of technical success, durable improvements in vessel patency, and few complications in most patients [8,10,12,13,14]. Stenting is also reported to reduce leg swelling, leg pain, and improve ulcer healing in the context of MTS [8,10].

Lymphedema is a common and morbid occurrence in MTS patients, but the true prevalence in this population is currently unknown. Despite improvements in some symptoms of lymphedema, the impact of iliac stenting on established lymphedema or lymphedema progression is currently unclear. In early stages of lymphedema (stages 0–I), fluid accumulation may be reversible, but later stages (stages II–III) are irreversible. Once initiated, the architectural tissue changes in lymphedema, such as adipocyte proliferation, fibrosis, and scar contracture, are progressive and debilitating [15].

Lymphedema can be effectively treated with appropriate non-invasive and surgical interventions [16]. In this study, we treated patients according to our previously published clinical algorithm [17]. Surgical interventions commonly include liposuction, lymphovenous anastomosis (LVA), and vascularized lymph node transfer (VLNT). The body of the literature on lymphedema and its management in MTS patients is sparse. Given the cryptic nature of MTS and the difficulty of diagnosis, patients may experience lymphedema progression for years before being diagnosed and effectively treated. At present, only two cases of physiologic surgical lymphedema management in MTS are reported in the literature [18].

Here, we present 26 cases of lymphedema in MTS managed with various combinations of LVA, VLNT, liposuction, and combination surgeries. These interventions may provide important clinical benefits to MTS patients with concomitant lymphedema, beyond what is gained from iliac stenting alone. Our findings present the largest case series to date. Surgical procedures were associated with reductions in excess limb volume in selected patients who had persistent lymphedema after venous and conservative management. Our findings carry clinical implications for treatment algorithms and screening practices.

## 2. Methods

Retrospective case series. Patients with diagnosed May–Thurner syndrome and concurrent left lower extremity lymphedema were retrospectively identified at a single large academic medical center between 1 January 2016 and 31 December 2025. Inclusion criteria comprised adults with MTS, lymphedema, and left lower extremity lymphedema. Patients with MTS without lymphedema, or that had lymphedema only in an unrelated area such as the upper extremities, were excluded. All eligible patients treated during the study period were included. All patients underwent stenting, which will hereafter be referred to as stenting and not as surgery; only lymphatic surgery will be referred to as surgery. Lymphedema was classified according to the International Society of Lymphology (ISL) stages. Lymphedema treatment was based on clinical evaluation according to the senior author’s previously published algorithm [17].

Surgical interventions included LVA, liposuction, VLNT, or a combination of those procedures, based on their indication. Nonsurgical intervention included CDPT, which included compression therapy using stockings or pneumatic compression devices, and manual lymphatic drainage in addition to patient education on skin care and exercise. CDPT was done 3 times a week for 6 months by certified lymphedema therapists. Pneumatic compression devices were recommended if the patient could obtain them. Stage I lymphedema patients were given Class 2 circular knit stockings (20–30 mmHg), stage II were given Class 3 circular knit stockings (30–40 mmHg), and stage III were given Class 3 flat knit stockings (40–50 mmHg). Adherence was assessed per patient and lymphatic therapist reports. If any patients underwent CDPT prior to surgery, they received standardized treatment based on CDPT frequency and duration, and compression garment use as above. All patients who underwent lymphatic surgery underwent CDPT postoperatively for 3 months, then maintained a standardized home-based care plan with daily manual lymphatic drainage.

Various data were collected, such as patient demographics, timing of diagnosis, and venous stenting procedure details. Imaging data included pre- and postoperative photography, lymphoscintigraphy, magnetic resonance imaging (MRI), and magnetic resonance lymphangiography (MRL). MRL imaging was interpreted in the standard fashion by radiologists, who were not blinded to the patients. The presence of new lymphatic vessel formation was defined based on identification on postoperative MRI.

Pre- and postoperative limb circumferences in both unaffected and affected limbs were recorded. Limb circumferences were measured every 4 cm for lower extremities and the truncated cone formula was used to approximate total limb volume. Excess limb volume was calculated as the difference in limb volume from the “affected” left lower extremity to the “unaffected” contralateral extremity. Negative excess limb volumes should be interpreted as decreased limb volumes. Percent excess volume change refers to the aforementioned excess limb volume in the affected limb pre-intervention to post-intervention as a percentage change. Negative percent changes should also be interpreted as a decrease in volume from pre- to post-intervention, and positive percent changes for volume increase. Two experienced advanced practice practitioners measured limb circumferences for every patient, and to optimize inter-rater reliability, they first underwent standardized teaching and observation by the attending surgeon (senior author). The clinical staff were not blinded to the prior measurements or treatment. The first follow-up evaluations took place at postoperative day 1, 3, and 7 for VLNT, liposuction, and LVA, respectively. Afterward, appointments were scheduled for 3, 6, 9, 12, and 24 weeks postoperatively for all patients including CDPT. Time points for “final” limb measurements were the latest time point, at least 6 months postop.

Descriptive statistics were calculated using R (R Foundation for Statistical Computing, Vienna, Austria). Continuous variables were reported as mean ± standard deviation and categorical variables as counts and percentages. No formal hypothesis testing was performed.

## 3. Results

Twenty-six patients met the inclusion criteria of lymphedema with concurrent MTS. A total of 73% (n = 19) were female, and 100% of patients identified as Caucasian (Table 1). The average age at MTS diagnosis was 41.92 years and average BMI was 23.65 ± 3.32. Lymphedema on average was diagnosed at age 42.72 ± 16.22 (Table 1). MTS was diagnosed based on clinical symptoms, i.e., left lower extremity swelling, pain, DVT and imaging (CT, MRI, venogram) confirmation of narrowing of left common iliac vein at least 50%. Criteria for stenting were set at a minimum of 60–70% stenosis in the presence of symptoms.

A total of 11% of the patients were ISL stage I, 34% were stage II and 53% were stage III. Lymphedema was definitively diagnosed an average of 0.8 years after MTS diagnosis. All patients underwent at least one iliac vein stenting procedure with the average time to first stenting of 1.18 years (SD = 3.46) after MTS diagnosis. All stents were patent at the time of lymphatic treatment, with 52% of patients reporting symptomatic improvement after stenting. Seven patients (26.9%) were managed with complete decongestive physical therapy (CDPT) alone: all three stage I patients (100%), two stage II patients (22.2%), and two stage III patients (14.3%). The remaining 19 patients (73.1%) underwent lymphatic surgery: seven of the nine stage II patients and 12 of the 14 stage III patients. Treatment allocation by stage is summarized in Table 2. The average follow-up period was 2.74 years. All patients included had follow-up of at least 6 months.

Five primary reasons were identified for pursuing lymphedema surgery following stenting and a period of conservative management with compression garments and/or pneumatic compression devices. Two patients developed recurrent cellulitis, prompting further surgical evaluation. Four patients experienced incomplete symptom resolution and gradual worsening despite conservative measures, which led them to seek surgical intervention. Five patients were lost to follow-up for several years, but ultimately returned for surgical consultation due to symptom non-resolution. Two patients were unable to maintain strict adherence to conservative therapy because of lifestyle limitations, resulting in worsening extremity edema. Finally, two patients achieved substantial limb volume reduction after stenting and conservative treatment, but desired further improvement through lymphatic surgery.

In a few patients, there was a prolonged interval between MTS diagnosis and definitive lymphatic surgery. The most significant outlier was a patient who remained stable for 23 years before developing recurrent cellulitis, which prompted surgical management 2 years later. Other outliers included patients who experienced a very slow progression of lymphedema over time and those who were lost to follow-up while managing other health issues.

Of the patients that underwent lymphatic surgery (n = 19), four patients had no improvement after stenting, and five had mild symptomatic improvement. Of the 19 patients who underwent lymphatic surgery, two (10.5%) underwent LVA alone, seven (36.8%) underwent liposuction alone, four (21.1%) underwent VLNT alone, and six (31.6%) underwent a combination of concurrent lymphatic procedures. Thirteen patients (68.4%) underwent one lymphatic procedure, five (26.3%) underwent two, and one (5.3%) underwent three or more. All percentages in this paragraph are calculated on the 19 surgical patients. The diagnosis of MTS did not alter the choice of the surgical procedure; this was guided by our previously published algorithm [17]. On average, LVA occurred 5.21 years after MTS diagnosis (SD = 2.37 years), VLNT occurred 6.75 years after MTS diagnosis (SD = 3.80 years), and the first liposuction procedure occurred 7.42 years after MTS diagnosis (SD = 6.54 years).

**Figure 1:**
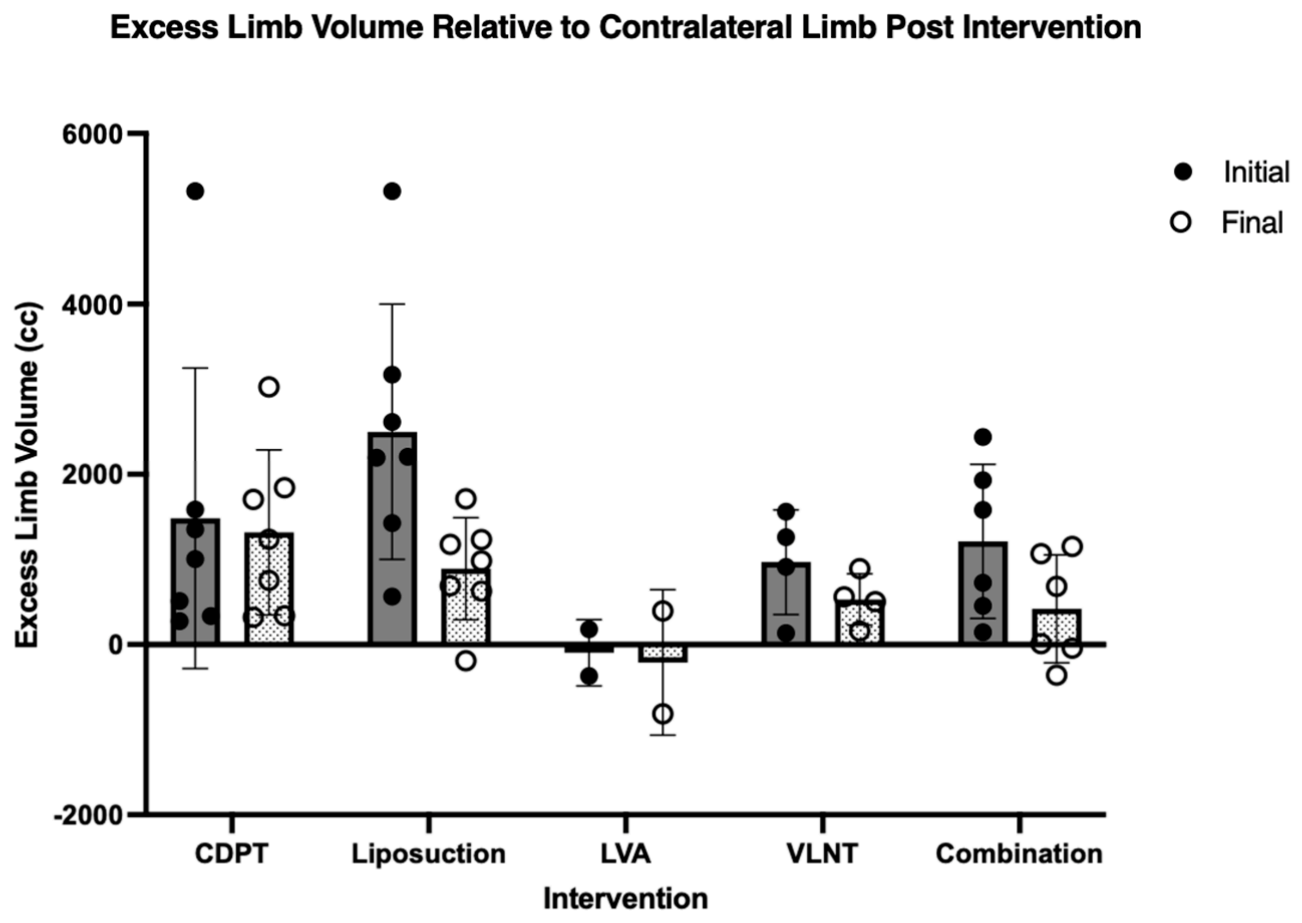
Average excess limb volume relative to contralateral limb post-intervention. Graph depicting initial and final excess limb volumes per intervention group. The error bars represent standard error.

All patients experienced an average volume decrease relative to the contralateral normal limb (Figure 1). Liposuction patients demonstrated the greatest absolute value magnitude in average relative volume reduction (1609.17 cc) and CDPT-only patients experienced the smallest volume decrease (114.79 cc).

**Figure 2:**
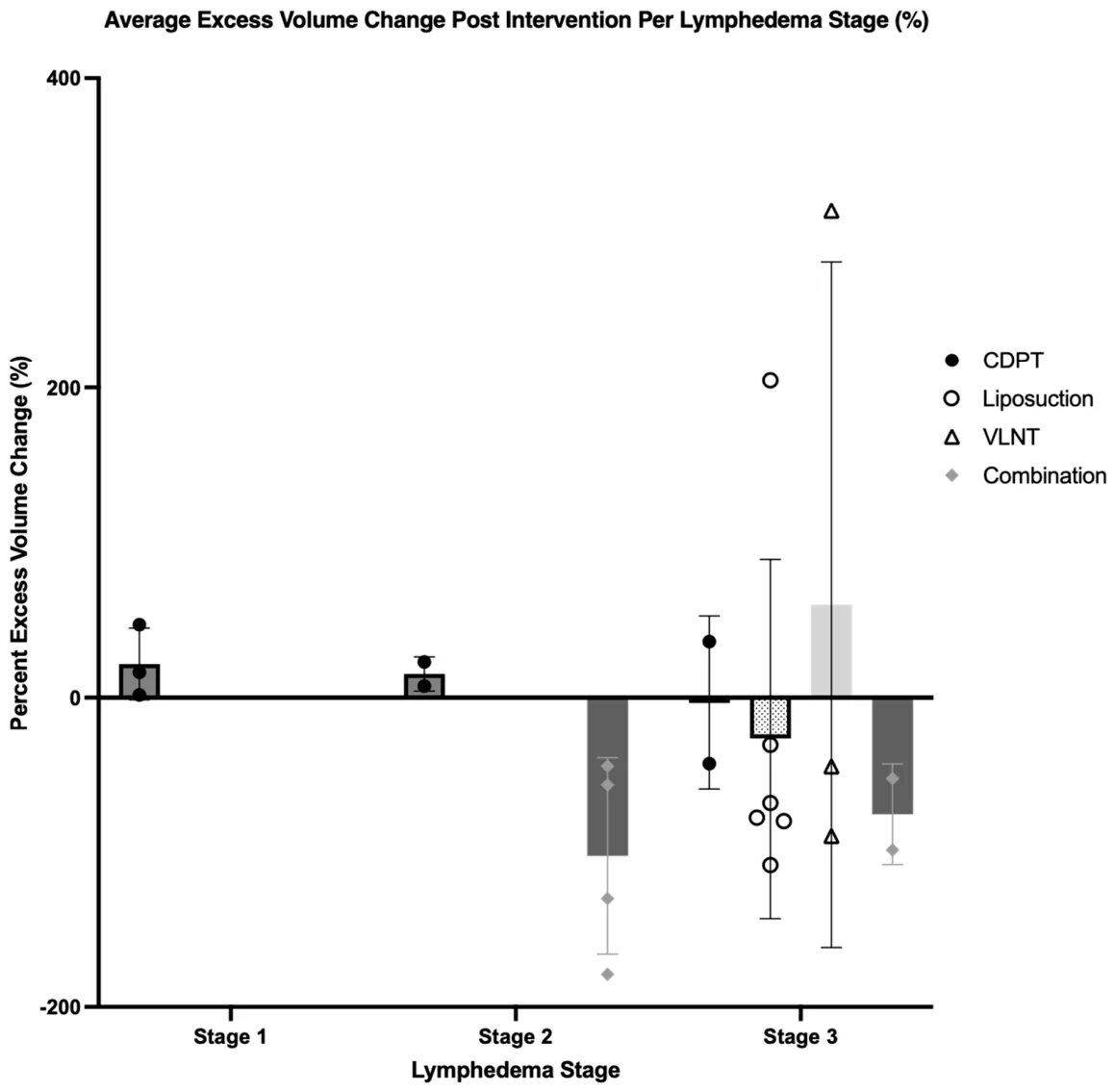
Average percent excess volume change post-intervention per lymphedema stage. Graph depicting the percent change after each intervention per stage. Positive percent change indicates volume increase, and a negative percent change indicates volume decrease, such as seen in limb shrinkage. Error bars represent standard error.

**Figure 3:**
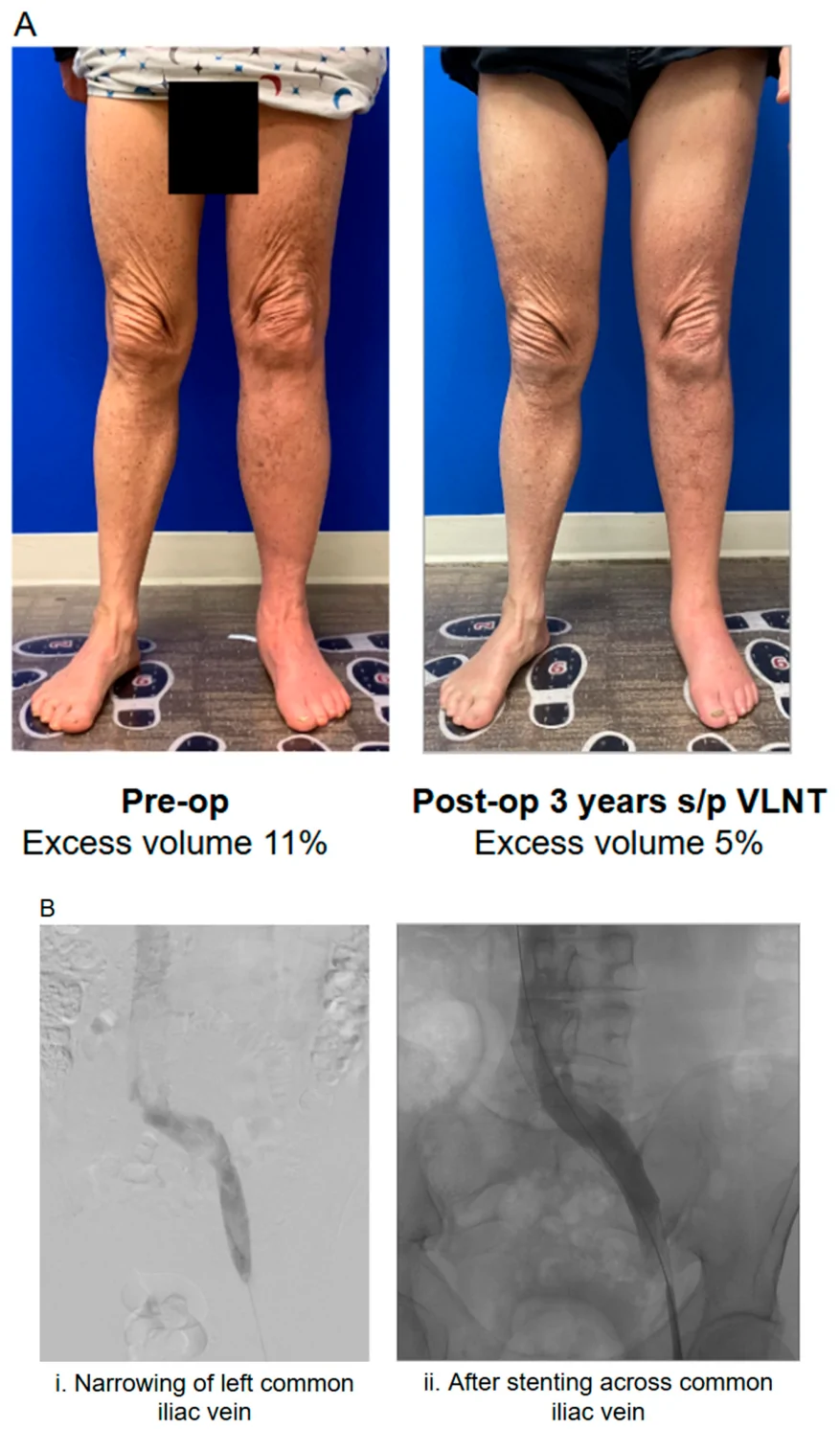

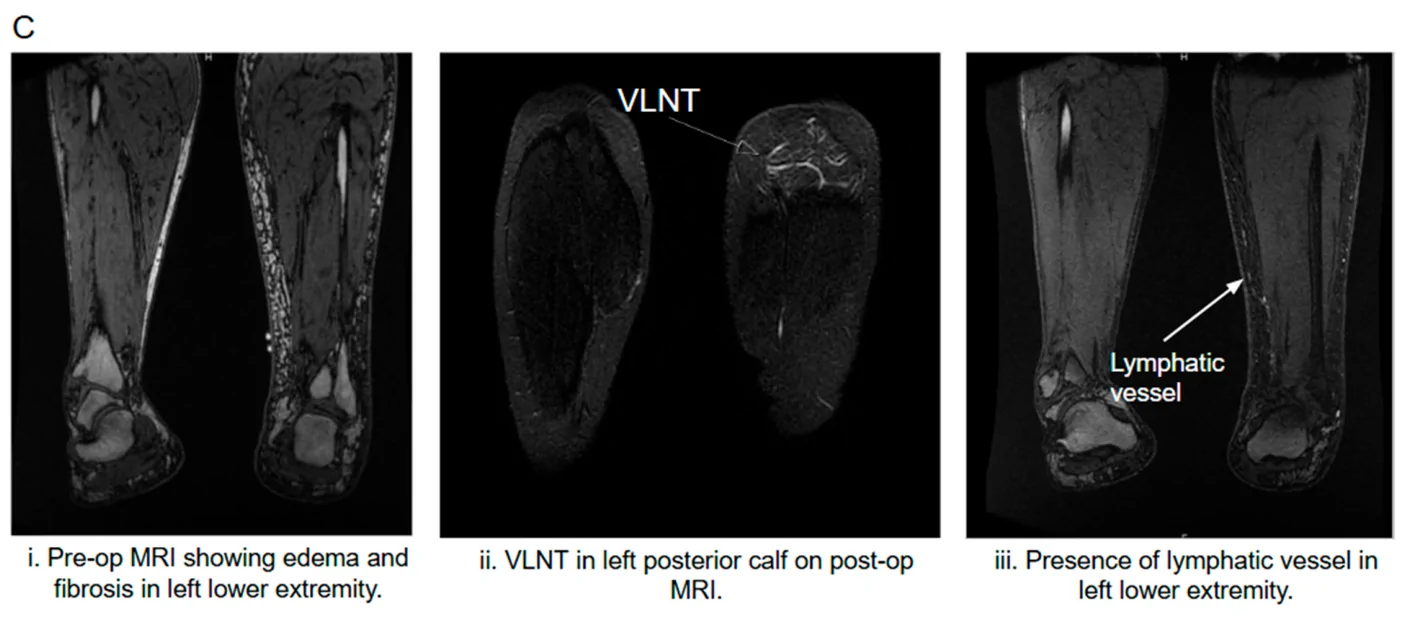
(**A**) A 69-year-old male with MTS (diagnosed in 2017) who presented with left leg lymphedema (dx 2019) and 11% excess volume in the left leg preoperatively. He underwent VLNT to the left calf that resulted in reduction in excess volume to 5% at years postop. (**B**) His venogram showed narrowing of the left common iliac vein (**i**) and after stenting to open the vein (**ii**). (**C**) Postoperative MRI at 1.5 y postop showed viable VLNT (**ii**) and presence of lymphatic vessel and minimal dermal backflow in the lower leg (**iii**) compared to preop, which showed edema and fibrosis in left lower extremity (**i**). Error bars represent standard error.

For groups with greater than one value per stage, average percent excess volume change was calculated (Figure 2). Stage I patients only underwent CDPT, and demonstrated an average volume increase of 21.74% ± 23.11. In the stage II group, CDPT patients experienced a percent volume increase of 15.26% ± 11.06, while combination patients experienced a 102% ± 63.31 volume decrease. Stage III patients comprised CDPT, liposuction, VLNT, and combination; these groups and two of three VLNT patients demonstrated decreased limb volume percentage. Twelve patients underwent pre- and postoperative MRI. Postoperative MRIs were done at 1–1.5 years after surgery, and had standard qualitative readings by radiology. Clinical findings and MRIs showed improved tissue bulk, decreased fibrosis, and edema reduction, as well as the formation of new lymphatic channels, as seen in Figure 3C, which is a scan from one patient that is representative of these findings.

All 19 patients who underwent lymphatic surgery were reviewed for postoperative complications. Three patients (15.8%) experienced a complication. Two patients (10.5%) developed delayed wound healing over the dorsum of the ankle following liposuction (one liposuction-only patient and one combination patient), both of which resolved with local wound care. One combination patient (5.3%) developed erythema at the VLNT recipient site on postoperative day 3 that was suspicious for superficial infection; this was treated with intravenous antibiotics with full resolution, and the patient subsequently underwent irrigation and debridement of the left leg wound. There were no episodes of flap loss, donor site morbidity or donor limb lymphedema, postoperative deep vein thrombosis or pulmonary embolism, or stent thrombosis.

## 4. Discussion

Our findings are consistent with the literature in terms of MTS patient demographics: generally adult white female patients [19]. We also observed an association with DVT history (15%), a common comorbidity in MTS [19]. Importantly, most patients present late in terms of disease severity for lymphedema treatment, which is associated with increased incidence of infection (40% = 8/20) [20]. Despite venous stenting and stent patency, extremity edema often persists. Late-stage lymphedema is characterized by chronic fibrofatty tissue deposition and fibrosis, which can damage fragile lymphatic vessels [21]. Proper lymphatic functioning depends on the high-pressure lymphatic vessels flowing into the low-pressure venous system [22]. An obstruction in the proximal venous drainage pipeline leads to backflow and subsequent venous regurgitation, damaging the delicate surrounding lymphatic system, leading to lymphedema progression.

Although MTS pathophysiology likely indicates the venous disease precedes lymphatic disease, 16 of 26 patients were first diagnosed with lymphedema, then later with MTS, despite the average age of lymphedema diagnosis being 0.8 years after their MTS diagnosis. This may be due to lack of awareness and clinical suspicion for MTS in early-phase disease. Our results indicate that despite optimal venous decompression via patent stenting, patients with MTS still have persistent lymphedema, which will require lymphatic surgical intervention. This has also been observed in numerous case studies [18]. In MTS patients that present with the chief complaint of lymphedema, we advise treating the venous disorder first before undergoing lymphatic surgery to address the possible venous component of the patients’ left lower extremity lymphedema. Based on our experience, this approach is more favorable; the patients are more likely to achieve symptomatic improvement and clinically relevant volume reduction after lymphatic surgery. Therefore, our proposed algorithm first optimizes venous treatment to improve outflow, e.g., via workup with ultrasound to rule out blood clots and vascular insufficiency and undergo stenting, then to address the lymphatic disorder.

In this series, surgical procedures were associated with reductions in excess limb volume in selected patients. MRI also confirmed decreased fibrosis and new lymphatic formation. CDPT patients in stage I and II experienced worsening lymphedema over time except for a modest 3.45% relative excess volume reduction in stage III. For LVA, we cannot conclude average efficacy based on the under-powered statistics, but of the two patients who underwent this procedure, one showed notable relative excess volume improvement (−370.88 to −814.83 cc). The other only slightly gained excess volume (from 3% to 6%), which was still within the 5% normal range of volume change. Liposuction was effective in reducing excess relative volume in the stage III patients. Stage II was not powered enough to truly conclude efficacy but the patient showed a 52% relative volume decrease. VLNT showed decreased percent relative volume in stage III and in two of three stage III patients. The combination surgical therapy consistently showed across stages that it was associated with the greatest excess volume reduction in our patient sample. However, conservative interventions may also induce physiological responses beyond changes in limb volume. Previous research in healthy women has reported short-term hematological and immune-related changes following repeated manual lymphatic drainage, although the clinical relevance of these findings in patients with established lymphedema remains to be determined [23].

Due to the strong association with lymphedema in MTS, opportunities for lymphedema prevention and earlier screening should be explored. We hypothesize that due to the lack of awareness or unknown disease prevalence, MTS is not routinely on the differential diagnosis list for patients presenting with unilateral lower extremity lymphedema. Additionally, since many patients initially present with late-stage lymphedema, the initial management is commonly focused on treatment and not further diagnosis for underlying vascular disease, which, as discussed above, may precede the lymphatic disease. Through this review, we aim to spread awareness of MTS, its strong association with lymphedema, and the utility of screening patients presenting with unilateral lower extremity lymphedema for MTS. For example, at the time of stenting, patients could undergo formal workup for lymphedema and be followed by the lymphedema team for early prevention. One team regularly screens patients with isolated lower extremity lymphedema for iliac vein compression since the true prevalence of MTS is unknown [18]. CTV has shown moderate utility in screening for patients presenting with isolated left lower extremity lymphedema and history suspicious for MTS [24]. Screening for cancer-related lymphedema has also begun to gain traction, implementing clinical assessment and limb measurement practices during patient visits [25].

As seen in our study, patients who did not receive surgical treatment trended toward continued progression of disease despite CDPT. Hence, early detection and intervention can potentially improve volume reduction, reduce tissue infection occurrence, and establish patient expectations ahead of time. Venous reflux in addition to obstruction cannot be fully corrected and will have comparatively worse outcomes than simple lymphatic obstruction [26]. Addressing the venous disorder first may lead to achieving volume normalization. The exact timeframe of lymphedema occurrence after MTS diagnosis is unknown, which highlights an opportunity for a prospective study to investigate if lymphedema may be preventable with early intervention.

## 5. Limitations

This study is limited by multiple factors, such as its retrospective, single-center design and small sample size with very small treatment subgroups that are under-powered to detect true statistical significance. We reported our trends based on our small volume of patients. Additionally, there was variable follow-up duration for the patients, but each had at minimum 6 months of postoperative visits and limb measurements. Outcome measures at least 6 months after surgery across all groups were used. Due to small sample size, there is not enough power to stratify further. Other limitations include limited ethnic diversity and heterogeneity of procedures. This study focuses on limb volume reduction, which is clinically relevant but does not fully represent treatment success; similarly, it does not include patient-reported outcomes, which is a future direction for our team. There was no directly comparable control group included in this case series, as it was designed to focus on patients who received lymphatic surgery or CDPT, not to compare outcomes against MTS patients with lymphedema who did not undergo any sort of lymphatic treatment. Importantly, treatment allocation was influenced by symptom severity, tissue fibrosis and adipose deposition, and suitability for specific surgical procedure or CDPT. Treatment allocation could also have been biased by patient preference. A potential confounder could be the treatment indication, due to different stages of lymphedema, which defined their treatment course. Further studies are needed to elucidate the true lymphedema prevalence in this population, more finely tease out details of lymphedema with and without femoral vein stenting, and provide more robust data on which lymphatic treatments are effective for each lymphedema stage in MTS patients. This study remains the largest published case series to date of MTS patients with concomitant lower extremity lymphedema that underwent lymphatic surgical intervention. Further research will aid in improving care for patients with MTS and lymphedema at large.

## 6. Conclusions

Lymphatic surgery is often an effective treatment for MTS-related lymphedema beyond what venous stenting can offer. Patients who did not undergo surgical treatment continued to have increasing limb volumes despite CDPT. Despite pathophysiology suggesting venous disease precedes lymphatic disease, some patients are diagnosed with lymphedema years before they are found to have underlying MTS. Many patients still have lymphedema after iliac vein stenting, which often responds well to lymphatic surgery. It is important to note these patients’ symptoms might be unrelated to MTS or were falsely diagnosed as MTS, and could be indicative of lymphedema not related to venous disease. However, the common co-occurrence of lymphedema and MTS presents an opportunity for screening, early intervention, and prospective studies.

## Figures and Tables

**Table 1:** Demographic and patient-related data.

		Male	Female	Total
		**n = 7 (26.9%)**	**n = 19 (73.1%)**	**n = 26**
Average BMI		25.57 ± 1.40 (Range 24–27)	22.95 ± 3.45 (Range 17–29)	23.65 ± 3.32 (Range 17–29)
Lymphedema Stage (ISL)	Stage I	n = 0 (0%)	n = 3 (15.79%)	n = 3 (11.54%)
	Stage II	n = 5 (71.43%)	n = 4 (21.05%)	n = 9 (34.62%)
	Stage III	n = 2 (28.57%)	n = 12 (63.16%)	n = 14 (53.85%)
History of trauma, surgery, or radiation		n = 3 (42.86%)	n = 10 (52.63%)	n = 13 (50%)
History of cellulitis		n = 2 (28.57%)	n = 7 (36.84%)	n = 9 (34.62%)
History of DVT		n = 2 (28.57%)	n = 2 (10.53%)	n = 4 (15.38%)
Average age at MTS Diagnosis (years)		41.92 ± 16.24 (Range 4–73)		
Average Age at Lymphedema Diagnosis (years)		42.72 ± 16.22 (Range 4–74)		
			Average Time to Intervention (years)	# of Patients with Intervention
Average Time to First Stent			1.18 ± 3.46 (Range 4–10)	n = 26 (100%)
Average Time to First Liposuction			7.42 ± 6.54 (Range 1–23)	n = 9 (34.62%)
Average Time to LVA			5-21 ± 2.37 (Range 3–8)	n = 7 (26.92%)
Average Time to VLNT			6.75 ± 3.80 (Range 3–14)	n = 10 (38.46%)

**Table 2:** Treatment allocation by International Society of Lymphology (ISL) stage. Percentages for CDPT alone and lymphatic surgery are calculated within each ISL stage; percentages in the procedure columns are calculated on the 19 patients who underwent lymphatic surgery. All patients, including those who underwent lymphatic surgery, received complete decongestive physical therapy; the CDPT alone column denotes patients managed conservatively without lymphatic surgery. CDPT, complete decongestive physical therapy; LVA, lymphovenous anastomosis; VLNT, vascularized lymph node transfer.

ISL Stage	Total, n	CDPT Alone, n (%)	Lymphatic Surgery, n (%)	LVA Only, n	Liposuction Only, n	VLNT Only, n	Combined, n
Stage I	3	3 (100%)	0 (0%)	0	0	0	0
Stage II	9	2 (22.2%)	7 (77.8%)	1	1	1	4
Stage III	14	2 (14.3%)	12 (85.7%)	1	6	3	2
Total	26	7 (26.9%)	19 (73.1%)	2 (10.5%)	7 (36.8%)	4 (21.1%)	6 (31.6%)

## Data Availability

The original contributions presented in this study are included in the article. Further inquiries can be directed to the corresponding author.
